# Isocitrate dehydrogenase inhibitors in acute myeloid leukemia

**DOI:** 10.1186/s40364-019-0173-z

**Published:** 2019-10-22

**Authors:** Xiaoyan Liu, Yuping Gong

**Affiliations:** Department of Hematology, West China Hospital of Sichuan University, No.37 Guo Xue Xiang, Chengdu, 610041 Sichuan Province China

**Keywords:** AML, IDH mutation, IDH inhibitor

## Abstract

Isocitrate dehydrogenase (IDH) is a key enzyme involved in the conversion of isocitrate to α-ketoglutarate (α-KG) in the tricarboxylic acid (TCA) cycle. IDH mutation produces a neomorphic enzyme, which can lead to the abnormal accumulation of R-2-HG and promotes leukemogenesis. IDH mutation occurs in 20% of acute myeloid leukemia (AML) patients, mainly including IDH1 R132, IDH2 R140, and IDH2 R172. Different mutant isoforms have different prognostic values. In recent years, IDH inhibitors have shown good clinical response in AML patients. Hence, enasidenib and ivosidenib, the IDH2 and IDH1 inhibitors developed by Agios Pharmaceuticals, have been approved by the Food and Drug Administration on 1 August 2017 and 20 July 2018 for the treatment of adult relapsed or refractory (R/R) AML with IDH2 and IDH1 mutations, respectively. IDH inhibitor monotherapy for R/R AML is efficacious and safe; however, there are problems, such as primary or acquired resistance. Clinical trials of IDH inhibitors combined with hypomethylating agents or standard chemotherapy for the treatment of R/R AML or newly diagnosed AML, as well as in post hematopoietic stem cell transplantation as maintenance therapy, are ongoing. This article summarizes the use of IDH inhibitors in AML with IDH mutations.

## Introduction

Approximately 40–45% of young and 10–20% of elderly AML patients treated with standard therapy will be cured. There are no more than 10% of relapsed or refractory (R/R) AML [1–4]. Advancements in next-generation sequencing (NGS) have increased our understanding on genomic and epigenomic landscapes in AML [5, 6], and therefore, precision therapy has become possible in AML treatment, multiple studies of targeted drugs (such as IDH inhibitors, FLT3 inhibitors and BCL-2 inhibitors, etc.) are ongoing [7–12]. Isocitrate dehydrogenase (IDH), converts isocitrate to α-ketoglutarate (α-KG) and has three different isoforms. IDH1 and IDH2 are located in the cytoplasm and mitochondria, respectively, while IDH3 is located in the mitochondrial matrix [13]. Recurring IDH1 and IDH2 mutations were reported in glioma in 2008 and 2009, respectively [14, 15], and later in AML, cholangiocarcinoma, chondrosarcoma, chondromas, and so on [16–18]. To date, no study has reported cancer-associated mutations in IDH3, although IDH3 has a similar function as that of IDH1 and IDH2.

IDH mutations occur in the active catalytic sites of the arginine residues resulting in a neomorphic enzyme activity converting α-KG to the oncometabolite R-2-hydroxyglutarate (R-2-HG) [19, 20]. The abnormal accumulation of R-2-HG affects multiple α-KG-dependent dioxygenases including abnormal methylation of histones and DNA [21–24], blocking cell differentiation. Because of the second mutation, R-2-HG can promote leukemogenesis [25–27], although the process is reversible [28]. IDH mutations were found to be stable during the evolution of AML with an incidence rate of 20% [29–32]. In AML, heterozygous IDH mutations are noted including IDH1 R132H/C, IDH2 R140Q, and R172K [19]. The incidences of IDH1 and IDH2 mutations are equivalent and mutually exclusive [19, 33–36]; however, the incidence rate of IDH2 R140Q was found to be higher than that of IDH2 R172K (9.2% vs. 2.9%) [35]. IDH mutations are mostly found in the intermediate-risk karyotype group, especially in the normal karyotype, on the other hand, IDH mutations always accompanied with NPM1 mutation [16, 29, 36], but is mutually exclusive with the TET2 mutation [37]. Further, IDH2 R172 and NPM1 mutations were not detected in the same patient samples [29, 38]. As shown in Table 1, different mutation isoforms have different prognostic values in AML, and remain controversial [29–31, 36, 39–47]. Some AML patients with IDH mutation, especially IDH2 R172 mutation, have a poor response to traditional chemotherapy and have a higher relapse rate [42]. Therefore, individualized treatment, especially targeted therapy for IDH mutations, may be an important option for such patients. In recent years, IDH inhibitors have shown good clinical response in AML patients. Based on phase 1/2 clinical trials, enasidenib and ivosidenib have been approved by the Food and Drug Administration (FDA) on 1 August 2017 and 20 July 2018 for the treatment of adult R/R AML with IDH2 and IDH1 mutations, respectively [48, 49]. This article provides a summary of the use of IDH inhibitors in AML with IDH mutations.

**Table 1:** The treatment response and prognostic value of IDH mutations in adult AML patients

Reference		% (Mut/All)	Common co-mutation	CR + CRi (%)	RR	OS	RFS	Prognostic value	Disease
[29]	IDH2	12.1 (54/446)	–	72.2	–	12y-OS, median not reached	–	Favorable	AML
[30]	IDH1	5.5 (27/493)	–	72.4	–	–	–	No value	AML
[39]	IDH1 R132	8 (107/1333)	NPM1+	81	10y: 55%;	10y: 34%	10y: 30%	No value	AML
[42]	IDH2 R140	8 (119/1473)	NPM1+	88	5y: 28%, 10y: 35%	5y: 57%, 10y: 46%	–	Favorable	AML
[42]	IDH2 R172	2 (29/1473)	None	48	5y: 70%, 10y: 70%	5y: 24%, 10y: 9%	–	Advers	AML
[44]	IDH1 R132	16 (34/213)	NPM1+	76	5y: 64%	4y: 46%	–	No value	CN-AML
[44]	IDH2 R172	6 (12/205)	None	58	5y: 100%	4y: 0%	–	Poor	CN-AML
[45]	IDH1 R132	14 (49/358)	NPM1+	73	–	3y: 29%	–	No value	CN-AML
[45]	IDH2 R140	16 (56/358)	NPM1+	70	–	3y: 39%	–	No value	CN-AML
[45]	IDH2 R172	4 (13/358)	None	38	–	3y: 0%	–	Poor	CN-AML
[46]	IDH1 R132	7.5 (32/426)	–	91	–	Median-OS: 20.9%	–	poor	AML
[46]	IDH2 R140	7.3 (31/426)	–	74	–	Median-OS: 35.8%	–	–	AML
[46]	IDH2 R172	2.6 (11/423)	–	100	–	Median-OS: 41.1%	–	Favorable	AML
[47]	IDH2 R172	1 (18/1540)	None	–	–	8y-OS: about 60%	–	Favorable	AML

*CR* complete remission, *CRi* CR with incomplete hematologic recovery, *OS* overall survival, *RFS* relapse-free survival, *RR* relapse rate, *CN-AML* cytogenetic normal AML

### IDH2 inhibitors

#### AGI-6780

AGI-6780 is the first small selectively allosteric inhibitor of IDH2 R140Q mutation developed by Agios Pharmaceuticals in 2013. It could reverse the abnormal methylation of histones and DNA, and reduce the 2-HG level that is caused by the mutant IDH2 R140Q in TF-1 cell lines [50], as well as in primary AML cells, thus leading to the differentiation of AML cells [51]. Lack of in vivo test along with good clinical response with the subsequently developed drug enasidenib limited the clinical application of AGI-6780 although it could reduce the abnormal 2-HG level, reverse cell phenotypic changes, and induce differentiation of the primary AML cells in vitro.

#### Enasidenib (AG-221, Idhifa®)

Enasidenib (Idhifa®) is an oral selectively allosteric inhibitor of IDH2 mutation developed by Celgene Corporation under a global exclusive license from Agios Pharmaceuticals. It shows more powerful inhibitory effects on IDH2 R172K rather than IDH1 R140Q (ORR, 53.3% vs. 35.4%; CR, 24.4% vs. 17.7%) mutation, although it has inhibitory effects on both the mutations [52]. FDA has approved enasidenib for the treatment of adult R/R AML with IDH2 mutations on 1 August 2017. The recommended oral dose is 100 mg once daily for at least 6 months or until disease progression or intolerable adverse reaction [53].

Enasidenib could reduce the serum total 2-HG level by more than 90%, reverse abnormal epigenetic changes, and induce differentiation of mutant IDH2 AML cells both in vitro and in murine xenograft models. Furthermore, it shows no cytotoxicity [54]. The results from a phase 1/2 clinical trial (NCT01915498) in adult R/R AML have shown that enasidenib monotherapy in adult R/R AML is efficacious and safe. When patients were administered with oral enasidenib once daily for 28 days in a cycle, an overall response rate (ORR) was 40.3%. About 87.3% of the patients reached their first treatment response within 5.8 months. The total CR rate was 19.3% with a median overall survival (OS) of 9.3 months and a median effect-free survival of 6.4 months, moreover, patients who reached CR had a longer OS of 19.7 months. Enasidenib seemed to induce AML cell differentiation and showed no cytotoxicity. Treatment-emergent adverse events were mainly elevated bilirubin (81%), nausea (50%), diarrhea (43%). decreased appetite (34%), and vomiting (34%). The treatment-related grade ≥ 3 adverse events occurred in 5% of the patients, and the most common were the elevation of indirect bilirubin level, nausea, differentiation syndrome (DS), tumor lysis syndrome, etc. However, with the continued use of the drug, the incidence of adverse reactions was gradually reduced. IDH-inhibitor-associated differentiation syndrome (IDH-DS) was reported in patients treated with enasidenib (IDH-DS of any grade occurred in 14% of patients, grade ≥ 3 occurred in 7% of patients), and perhaps the myeloid differentiation was the reason. All-trans retinoic acid (ATRA)-related DS occurred in acute promyelocytic leukemia (APL) with the incidence of 2–31%, It was thought that inflammatory cytokines and rapidly infiltrated by mature cells lead to the DS, although the pathophysiology of ATRA-related DS is not entirely understood [55]. It remains unclear whether IDH-DS has the same mechanism with the DS occurred in APL, although they have the similar incidence, clinical manifestation, and both could be cured with glucocorticoids, hydroxyurea, and diuretics [56]. A phase 3 randomized, open-label study comparing the efficacy and safety of AG-221 vs. conventional care regimens (CCR) in older R/R AML patients (≥ 60 years) with an IDH2 mutations is presently ongoing (NCT02577406). The initial results have shown that the median OS in the enasidenib group was longer than that in the CCR group (8 months vs. 5 months) [57].

Enasidenib combined with standard chemotherapy or the hypomethylating agent (HMA) azacytidine is well tolerated and effective in AML patients with IDH2 mutation. The initial results of an ongoing clinical trial of enasidenib combined with the standard induction chemotherapy (NCT02632708) in the treatment of newly diagnosed AML patients with IDH2 mutations showed that the ORR was 62%, the CR rate was 50%, and the less 30-days and 60-days mortality rate were 5 and 8%, respectively [58, 59]. Azacytidine and IDH inhibitors can directly or indirectly reduce DNA methylation levels and have synergistic effects on inducing cell differentiation. These characteristics have inspired an ongoing phase 1/2 clinical trial of the combination of the two agents (NCT02677922), and the initial results indicated an ORR of 50% (3/6), one patient was withdrawn because of disease progression, while two others are still in a stable state [60]. A phase 2 clinical trial of enasidenib combined with azacytidine for the treatment of R/R AML with IDH2 mutations (NCT03683433) and a phase 1 clinical trial of enasidenib as maintenance therapy in post-HSCT AML patients with IDH2 mutations (NCT03728335) are ongoing. The ongoing clinical trials of enasidenib in adult AML are shown in Table 2.

**Table 2:** Ongoing Clinical trials of enasidenib in adult AML

NCT Number	Phase	disease	Interventions	Status
NCT03683433	Phase 2	R/R AML;	Enasidenib plus Azacitidine	Recruiting
NCT03881735	Phase 2	R/R AML;	Enasidenib;	Not yet recruiting
NCT03825796	Phase 2	R/R AML	Enasidenib plus CPX-351	Recruiting
NCT01915498	Phase 1/2	Advanced AML	enasidenib	Active, not recruiting
NCT03013998	Phase 1/2	Previously Untreated AML	enasidenib	Recruiting
NCT02632708	Phase 1	Newly Diagnosed AML	Enasidenib plus standard chemotherapy	Active,not recruiting
NCT02677922	Phase 1/2	Newly Diagnosed AML	Enasidenib plus Azacitidine	Active,not recruiting
NCT03839771	Phase 3	Newly Diagnosed AML;	AG-221 plus standard chemotherapy	Not yet recruiting
NCT03515512	Phase 1	Post-HSCT AML	Enasidenib as maintenance therapy after HSCT	Recruiting
NCT03728335	Phase 1	Post-HSCT AML	enasidenib as maintenance therapy after HSCT	Not yet recruiting
NCT02577406	Phase 3	AML ≥60 years	AG-221 versus conventional care regimens	Recruiting

CPX-351:liposome-encapsulated daunorubicin-cytarabine

### IDH1 inhibitors

Different IDH1 inhibitors are effective against different types of IDH1 mutation. The ongoing clinical trials of IDH1 inhibitors in adult AML are shown in Table 3.

**Table 3:** Ongoing clinical trials of IDH1 inhibitors in adult AML

NCT Number	Phase	disease	Interventions	Status
NCT04013880	Phase 1/2	R/R AML	FT-2102 plus ASTX727	Not yet recruiting
NCT02719574	Phase 1/2	AML	FT-2102 as single agent; FT-2102 plus azacitidine/ cytarabine;	Recruiting
NCT03471260	Phase 1/2	R/R AML	Ivosidenib plus Venetoclax with or without azacitidine	Recruiting
NCT02074839	Phase 1	R/R AML; Untreated AML;	ivosidenib	Active, not recruiting
NCT02677922	Phase 1/2	Newly Diagnosed AML	Ivosidenib plus Azacitidine	Active, not recruiting
NCT03173248	Phase 3	Untreated AML	Ivosidenib plus azacitidine vs placebo plus azacitidine	Recruiting
NCT02632708	Phase 1	Newly Diagnosed AML	Ivosidenib plus standard chemotherapy	Active, not recruiting
NCT03839771	Phase 3	Newly Diagnosed AML	Ivosidenib plus standard chemotherapy	Recruiting
NCT03013998	Phase 1/2	Previously Untreated AML	ivosidenib	Recruiting

ASTX727 is an oral deoxyribonucleic acid (DNA) methyltransferase (DNMT) inhibitor

#### Ivosidenib (AG-120, Tibsovo®)

The unacceptable pharmacodynamic and pharmacokinetic properties have limited the clinical applications of AGI-5198, and AG-120 was the drug developed after due optimization [61]. Ivosidenib (Tibsovo®) is a small molecule developed by Agios Pharmaceuticals and can be orally administered. It is an inhibitor of mutated IDH1 and could reduce the total serum 2-HG level and induce mutated IDH1 AML cell differentiation. It was approved by the FDA for the treatment of adult R/R AML with IDH1 mutations on 20 July 2018. The recommended dose is 500 mg orally once daily (the dose can be adjusted according to the patient’s condition) until disease progression or occurrence of unacceptable toxicity or HSCT [49].

The approval of ivosidenib by the FDA was based on a phase 1/2 clinical trial of ivosidenib monotherapy in adult R/R AML with IDH1 mutations (NCT02074839). The trial concluded that ivosidenib monotherapy was safe and effective and it could reduce or even eliminate variant allele frequency (VAF) so that some patients could achieve a deep and lasting remission. In this clinical trial, the ORR was 41.6% with a median response time of 6.5 months, and the CR rate was 21.6% with a median duration time of 9.3 months. The patients who reached molecular remission had a longer CR duration than those who did not. The CR + CRi (CR with incomplete hematologic recovery) rate was 30.4%, thus leading to 35% of the patients being independent of blood transfusion. Ivosidenib induce AML cell differentiation and seems no cytotoxicity. Adverse events occurred in 99% patients treated with ivosidenib. Mainly included diarrhea (31%), leukocytosis (30%), febrile neutropenia (29%), nausea (28%), fatigue (26%), dyspnea (25%), prolonged QT interval (25%), peripheral edema (22%), anemia (22%), fever (21%) and cough (21%). There were no adverse events related deaths, although the treatment-related grade ≥ 3 adverse events occurred in 21% of patients, and the most common were prolonged QT interval, DS and leukocytosis, which were also the most common reasons for drug withdrawal. Treatment with hydroxyurea or leukapheresis could be initiated for leukocytosis. IDH-DS occurred in about 11% of the patients with a median onset time of 29 days, and could be cured by temporary withdrawal of the drug and adding glucocorticoids and diuretics [34, 62, 63].

The ongoing clinical trial of ivosidenib combined with standard therapy or azacytidine for the treatment of adult R/R AML showed promising results. Ivosidenib combined with standard therapy was safe and effective in both primary and secondary AML (NCT02632708), although the ORR was higher in the former (86% vs. 44%) [59]. The CR rate was 3/5 (the other two patients had a stable disease) in a clinical trial of ivosidenib combined with azacytidine for the treatment of AML (NCT02677922) [60].

A global phase 3 clinical trial evaluating the efficacy and safety of ivosidenib combined with azacytidine vs. placebo combined with azacytidine in newly diagnosed AML patients with IDH1 mutations who are not suitable for intense chemotherapy is ongoing (NCT03173248). A phase 1/2 clinical trial of ivosidenib combined with venetoclax with or without azacytidine is also ongoing (NCT03471260).

#### BAY1436032

BAY1436032 is an orally administered allosteric inhibitor of all the IDH1 mutation proteins. It can pass through the blood-brain barrier [64]. BAY1436032 monotherapy can reduce the 2-HG level to a maximum, inhibit the self-renewal and proliferation of leukemia stem cells, and reverse the abnormal methylation of histone in patient-derived xenograft (PDX) mouse models [65]. BAY1436032 combined with azacytidine induced AML cell differentiation by regulating the EGR-GFI1-NFκB pathway [66]. A single arm non-randomized, multicenter phase 1/2 clinical trial of BAY1436032 for the treatment of advanced AML has been completed, but the results have not been reported yet (NCT03127735).

#### FT-2102

FT-2102 is an inhibitor of the mutated IDH1. A phase 1/2 clinical trial of FT-2102 monotherapy and combined therapy with azacytidine or cytarabine for the treatment of adult R/R AML is ongoing (NCT02719574). The phase 1 results indicated the efficacy and safety of the drug. The ORR was 33% and the CR rate was 14%, and there were no deaths related to FT-2102. The main adverse reactions were thrombocytopenia, febrile neutropenia, anemia, pneumonia, etc. There were no deaths related to FT-2102. Like for other IDH inhibitors, IDH-DS was reported, but it could be cured by withdrawing the drug and adding dexamethasone and hydroxyurea. Based on the phase 1 data, FT-2102 at a dose of 150 mg twice daily has been administered in the ongoing phase 2 clinical trial [67]. In addition, a phase 1/2 clinical trial of FT-2102 combined with DNA methyltransferase (DNMT) inhibitor for the treatment of R/R AML with IDH R132 mutation is presently ongoing (NCT04013880).

#### IDH305

IDH305 is a selective allosteric inhibitor of IDH1 mutation. It is an optimized product of IDH889. Preclinical studies have indicated that it could reduce the 2-HG level and induce differentiation of AML cells [68]. A phase 1 clinical trial of IDH305 for the treatment of advanced malignancies with IDH R132 is ongoing (NCT02381886). The initial results have revealed a good clinical effect. The ORR, CR rate, Cri, and PR rate were 33% (7/21), 9.5% (2/21), 4.8% (1/21), and 19%, respectively. The adverse events were mainly raised bilirubin and lipase [69]. Further studies evaluating the safety and tolerability of IDH305 as a single agent and in combination were needed.

#### Other IDH1 inhibitors

The other IDH1 inhibitors include AGI-5198, ML309 (AGI-5027), GSK 321, and DC_H31. AGI-5198 is a selective IDH1 R132H inhibitor [70], The pharmacokinetic properties of this drug (rapid metabolism and clearance) have limited its research and application value [64]. ML309 and GSK 321 have been mainly tried in solid tumors at present, although both are selective inhibitors of the mutant IDH1 [35, 71]. Recently, a new small molecular allosteric inhibitor of IDH1 R132H/C, called DC_H31, has been developed. It could reduce the 2-HG level and inhibit cell proliferation and promote cell differentiation in HT1080 cell lines [72], However, in vivo trials are needed to validate these effects to develop this drug as a promising new targeted inhibitor shortly.

### Pan-IDH inhibitor AG-881

Developed by Agios Pharmaceuticals, AG-881 is a pan-IDH inhibitor that shows inhibitory effects to both IDH1 and IDH2 mutations. The mutated IDH1 and IDH2 bind to AG-881 at different residues in a similar way due to different allosteric inhibitory regions. AGI-881 combined with IDH1 R132H and IDH2 R172K acts faster than IDH2 R140. The drug could easily pass through the blood-brain barrier and induce myeloid differentiation, thereby making it a more promising IDH inhibitor [73, 74]. A phase 1 clinical trial evaluating the efficacy and safety of AGI-881 in myeloid malignancies with mutated IDH1/IDH2 is presently ongoing (NCT02492737).

### Indirect IDH inhibitors

#### Venetoclax

Abnormal accumulation of R-2-HG inhibits cytochrome c oxidase (COX) producing an environment similar to hypoxia, and thus, activates the apoptotic precursor proteins BAX and BAK through the BCL-2 homologous effector. The mitochondrial outer membrane becomes transparent and ultimately apoptosis is promoted. However, the anti-apoptotic gene BCL-2 could antagonize BAX and BAK, thereby preventing the increased permeability of the mitochondrial outer membrane [75]. This promotes cell survival, and the mutated IDH cells show dependence on BCL-2 [76, 77]. Venetoclax is a highly selective BCL-2 inhibitor. Venetoclax monotherapy in IDH1/IDH2 mutated AML patients led to a CR + CRi up to 33% [78]. However, when combined with decitabine or azacytidine, the CR + CRi were 33 and 59% in mutated IDH R/R AML patients and AML patients not suitable for intensive treatment, respectively [76, 79]. When venetoclax was combined with decitabine or azacytidine for the treatment of older AML patients, the CR + CRi was 71% with a median OS of 24.4 months [80]. Therefore, venetoclax is a promising new drug for the treatment of AML.

#### Inhibitors of glutaminase and glutamate dehydrogenase (GLUD)

^13^C-tracing studies have shown that the carbon atoms of 2-HG in AML cells with IDH mutations are mainly derived from glutamine or glutamate. Glutamine is converted to glutamate catalyzed by glutaminase, and then GLUD catalyzes the oxidative decarboxylation of glutamate to produce α-KG. Finally, α-KG is converted to 2-HG in the mutated IDH cells [81, 82]. The inhibitors of glutaminase could jeopardize the metabolism of glutamine as a consequence of selective inhibition of the proliferation of the IDH mutant AML cells in vitro [83]. In the HCT116 colorectal cancer cells, the use of glutamate dehydrogenase inhibitor epigallocatechin-3-gallate EGCG could reduce the 2-HG level and inhibit the proliferation of the IDH mutant cells [82]; however, this should be further verified in AML cell lines.

#### Target of HIF-1α

In IDH mutant cells, the abnormal accumulation of R-2-HG could down-regulate the levels of HIF-1α by activating proline 4-hydroxylase EgIN as a result of inhibition of cell differentiation. This may be one of the factors driving leukemogenesis [28, 84–86]. Thus, in the IDH mutant cancer cells, the target of HIF-1α may be a new treatment choice for AML therapy [27].

### Resistance to the IDH inhibitors

There were some problems in the clinical applications of the IDH inhibitors in AML patients with mutations, such as primary and acquired resistance, although IDH inhibitors could provide in these patients an average survival time of 18–21 months [87]. Among patients who had primary resistance, there were some mutations in the NRAS and MAPK pathways, which might be the causes of primary resistance [38, 54].

The mechanisms of acquired resistance remain unclear. One study found that in R/R AML patients with IDH2 R140Q mutation treated with enasidenib, there are second site IDH2 mutations in the trans-state [Q316E mutation (at glutamine 316 position) and I319M mutation (at isoleucine 319 position) when they had acquired resistance [88]. Another study found that two R/R AML patients with IDH1 R132C mutation had achieved CR after treatment with ivosidenib. The final relapse was because of the acquired resistance to ivosidenib, and the IDH2 R140Q mutation was detected with the relapse [89]. Likewise, IDH1 R132C or R132H mutations were detected in the mutant IDH2 AML patients who had acquired resistance to enasidenib [87]. In summary, we speculate that the second site mutations of the allele and the mutual conversion of IDH mutant subtypes are the two mechanisms leading to the acquired resistance to the IDH inhibitors.

## Conclusions

Enasidenib and ivosidenib have been approved by the FDA for the treatment of adult R/R AML with mutant IDH2 and mutant IDH1, respectively, due to their high treatment response and absence of cytotoxicity in clinical trials [48, 49]. However, there exist some problems, such as resistance. Hence, a combined therapy and the pan-IDH inhibitors may, to some degree, resolve the question of acquired resistance to IDH inhibitors [89]. Meanwhile, racial and ethnic disparities of IDH mutations should also be considered and studied [90]. IDH mutations are often accompanied by abnormal methylation of DNA, and hence, IDH inhibitors combined with demethylating agents may become one of the main therapeutic ways for the treatment of IDH mutant AML patients. Because IDH mutations are the early mutations in AML, therefore, the application of IDH inhibitors should not only be limited to R/R AML patients but also all AML patients having IDH mutations. Clinical trials of the IDH inhibitors combined with standard chemotherapy or HMA for the treatment of R/R AML or newly diagnosed AML are ongoing. Furthermore, clinical trials evaluating the clinical efficacy of IDH inhibitors as maintenance therapy in post-HSCT AML patients are also ongoing.

## Data Availability

Not applicable.

## Abbreviations

AML: Acute myeloid leukemia
APL: Acute promyelocytic leukemia
ATRA: All-trans retinoic acid
CN-AML: Cytogenetic normal AML
COX: Cytochrome c oxidase
CR: Complete remission
CRi: CR with incomplete hematologic recovery
DS: Differentiation syndrome
FDA: Food and Drug Administration
HMA: Hypomethylating agents
HSCT: Hematopoietic stem cell transplantation
IDH: Isocitrate dehydrogenase
ORR: Overall response rate
OS: Overall survival
PDX: Patient derived Xenograft
R/R AML: Relapsed or refractory AML
RFS: Relapse-free survival
RR: Relapse rate
TCA: Tricarboxylic acid cycle
VAF: Variant allele frequency
α-KG: α-ketoglutarate
